# Identification of the Biomarkers and Pathological Process of Osteoarthritis: Weighted Gene Co-expression Network Analysis

**DOI:** 10.3389/fphys.2019.00275

**Published:** 2019-03-19

**Authors:** Hui-Yun Gu, Min Yang, Jia Guo, Chao Zhang, Lu-Lu Lin, Yang Liu, Ren-Xiong Wei

**Affiliations:** ^1^Department of Orthopedic, Zhongnan Hospital of Wuhan University, Wuhan, China; ^2^Department of Plastic Surgery, Zhongnan Hospital of Wuhan University, Wuhan, China; ^3^Center for Evidence-Based Medicine and Clinical Research, Taihe Hospital, Hubei University of Medicine, Shiyan, China

**Keywords:** osteoarthritis, biomarkers, WGCNA, pathological process, hub genes

## Abstract

Osteoarthritis (OA) is a joint disease resulting in high rates of disability and low quality of life. The initial site of OA (bone or cartilage) is uncertain. The aim of the current study was to explore biomarkers and pathological processes in subchondral bone samples. The gene expression profile GSE51588 was downloaded from the Gene Expression Omnibus database. Fifty subchondral bone [knee lateral tibial (LT) and medial tibial (MT)] samples from 40 OA and 10 non-OA subjects were analyzed. After data preprocessing, 5439 genes were obtained for weighted gene co-expression network analysis. Highly correlated genes were divided into 19 modules. The yellow module was found to be highly correlated with OA (*r* = 0.71, *p* = 1e-08) and the brown module was most associated with the differences between the LT and MT regions (*r* = 0.77, *p* = 1e-10). Gene ontology functional annotation and Kyoto Encyclopedia of Genes and Genomes pathway enrichment indicated that the yellow module was enriched in a variety of components including proteinaceous extracellular matrix and collagen trimers, involved in protein digestion and absorption, axon guidance, ECM-receptor interaction, and the PI3K-Akt signaling pathway. In addition, the brown module suggests that the differences between the early (LT) and end (MT) stage of OA are associated with extracellular processes and lipid metabolism. Finally, 45 hub genes in the yellow module (COL24A1, COL5A2, COL3A1, MMP2, COL6A1, etc.) and 72 hub genes in the brown module (LIPE, LPL, LEP, SLC2A4, FABP4, ADH1B, ALDH4A1, ADIPOQ, etc.) were identified. Hub genes were validated using samples from cartilage (GSE57218). In summary, 45 hub genes and 72 hub genes in two modules are associated with OA. These hub genes could provide new biomarkers and drug targets in OA. Further studies focusing on subchondral bone are required to validate these hub genes and better understand the pathological process of OA.

## Introduction

Osteoarthritis (OA) is a prevalent, heritable degenerative joint disease, primarily involving large weight-bearing joints in the hip and the knee (Valdes and Spector, 2011; Ryd et al., 2015). As the most common musculoskeletal disease (Picavet and Hazes, 2003), OA causes major pain, disability, low quality of life and an increased social healthcare burden globally (Centers for Disease Control and Prevention [Cdc], 2009). A report (Murphy and Helmick, 2012) investigating 27 million adults found that OA was diagnosed in over 10% of adults and was the fourth most common cause for hospitalization. The World Health Organization Scientific Group estimated that OA caused health issues in 10% of the world population, over 60 years old (Woolf and Pfleger, 2003).

Osteoarthritis is characterized by a complex pathological process including progressive cartilage erosion, osteophyte formation, subchondral bone modification, and synovial inflammation. It involves a long disease span of approximately10 to 20 years from onset to the end-stage, when joint replacement is recognized as the most effective treatment (Chu et al., 2014). Therefore, better identification is urgently required. Biomarkers, which are biological molecules that indicate biological processes, could be used for the identification of OA. Previous studies have mainly focused on the gene expression profiles of articular cartilage, meniscus or synovium from progressive OA patients, and the use of the resulting biomarkers for progressive OA is widespread. However, few consensual biomarkers are available for subchondral bone. Currently, accumulative evidence shows that alterations in subchondral bone are associated with the initiation of OA (Burr and Gallant, 2012). An analysis of gene expression microarray profiling of subchondral bone samples could contribute to the identification of new biomarkers and increase the mechanistic understanding necessary to provide early prevention and management of OA (Vukusic et al., 2013).

Weighted gene co-expression network analysis (WGCNA), a new systems biology method, is increasingly being used in bioinformatics to analyze gene expression microarray profiling data (Stuart et al., 2003; Zhang and Horvath, 2005; Langfelder and Horvath, 2008). By constructing a gene co-expression network, highly correlated genes are clustered into several modules. After relating modules to external information, biologically interesting modules are detected. From interesting modules associated with important biological functions or pathways, critical genes are found which play key roles in the phenotype and the development of disease, such as those involved in body weight (Ghazalpour et al., 2006), brain cancer (Horvath et al., 2006), diabetes (Keller et al., 2008), and osteoporosis (Farber, 2010). In addition, WGCNA can be used to screen candidate biomarkers or therapeutic targets. Therefore, we conducted the current study to find new biomarkers, relevant genes or potential mechanisms associated with OA.

## Materials and Methods

### Data Collection and Preprocessing

The gene expression profile of OA was downloaded from the Gene Expression Omnibus database ^1^. The GSE51588 microarray dataset (Chou et al., 2013) was obtained from 50 subchondral bone samples including those from 10 non-OA control subjects (five from the lateral tibial plateau and five from medial tibial plateau) and 40 OA subjects (20 from the lateral tibial plateau and 20 from the medial tibial plateau). A series matrix file was preprocessed to find differentially expressed genes based on variance analysis and 5439 genes were obtained for subsequent analysis.

### Co-expression Network Construction

The “WGCNA” package (Langfelder and Horvath, 2008) in R software was used for the network construction. Samples which met the Z.K value < -2.5 were deemed as outlying and removed from the expression and trait data. The Pearson correlation coefficients were calculated for all gene comparisons. Then, a weighted network adjacency matrix was calculated based on a_ij_ = | cor (x_i_, x_j_)|^β^. X_i_ and x_j_ are the nodes i and j of the network and β was determined using a scale-free topology criterion (Zhang and Horvath, 2005). The adjacency matrix was converted to a topological overlap matrix to identify gene modules, clusters of highly interconnected genes. A topological overlap measure (TOM) was used to determine the network interconnectedness. Gene modules were detected using the hierarchical clustering method based on a TOM-based dissimilarity measure (1-TOM) (Ravasz et al., 2002). The identification of branches of a hierarchical clustering dendrogram was conducted through the dynamic branch cut method (Langfelder et al., 2008).

### Identification of Clinically Significant Modules and Functional Annotation

Modules from hierarchical clustering with a dense correlation with biological or clinical information were selected as the interesting modules for subsequent analysis. In this process, gene significance (GS), module significance (MS), and module eigengene (ME) were calculated. GS was defined as the minus log of a *p*-value and MS was the average gene significance across the module gene. ME was the first principal component of a given module. The significance between the ME and a clinical trait was also calculated, as modules with a high trait significance were associated with pathways and could be a candidate (Ghazalpour et al., 2006; Fuller et al., 2007). Gene ontology (GO) functional annotation and Kyoto Encyclopedia of Genes and Genomes (KEGG) pathway enrichment via the database for Annotation, Visualization, and Integrated Discovery (Dennis et al., 2003), were performed to further validate the selected modules by finding the underlying mechanism and biological pathways.

### Identification and Validation of Hub Genes

The hub genes of an interesting module were determined through an absolute value of the geneModuleMembership > 0.9 and a geneTraitSignificance > 0.2 (Horvath and Dong, 2008). All genes from the selected interesting modules were then mapped into a protein–protein interaction (PPI) network using the Search Tool for the Retrieval of Interacting Genes database (Szklarczyk et al., 2015). The PPI network was visualized using Cytoscape (Shannon et al., 2003) and genes with more than 10 nodes were chosen for the determination of common components between the interesting module and the PPI network. The gene expression profile GSE57218 from cartilage samples was used to validate some hub genes within the yellow and brown modules through a one-way analysis of variance (*p* < 0.05).

## Results

### Data Preprocessing and Co-expression Network Construction

**FIGURE 1:**
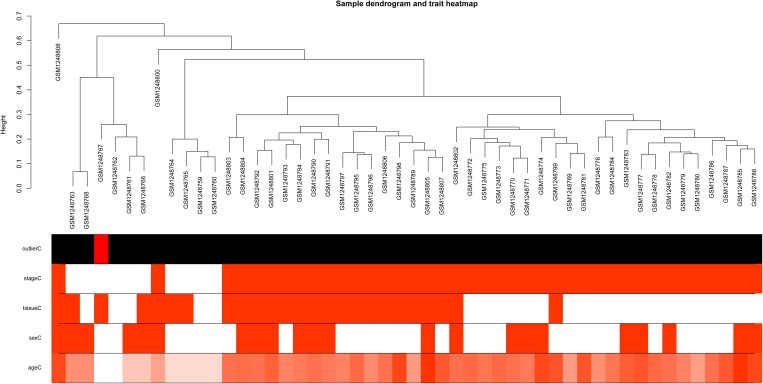
Sample dendrogram and trait heatmap. The leaves of the tree correspond to osteoarthritis (OA) samples and non-steoarthritis (non-OA) samples. The first color band underneath the tree indicates which arrays appear to be outlying. The second color band represents disease stages which indicate OA and non-OA (red indicates high values). The third color band represents tissue sources which indicate samples from knee lateral tibial (LT) and medial tibial (MT) (red indicates high values). Similarly, the remaining color-bands color-code the numeric values of physiologic traits.

**FIGURE 2:**
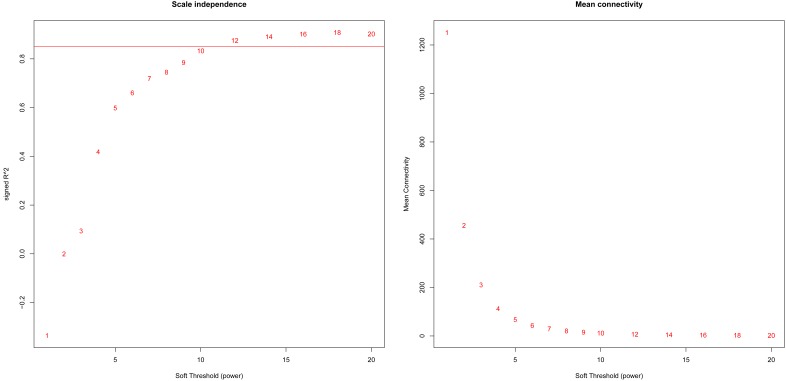
Analysis of network topology for various soft-theholding powers. The left panel shows the scale-free fit index, signed Rˆ^2^ (y-axis) and the soft threshold power (x-axis). β = 12 was chosen for subsequent analysis. The right panel shows the mean connectivity (y-axis) is a strictly decreasing function of the power β (x-axis).

**FIGURE 3:**
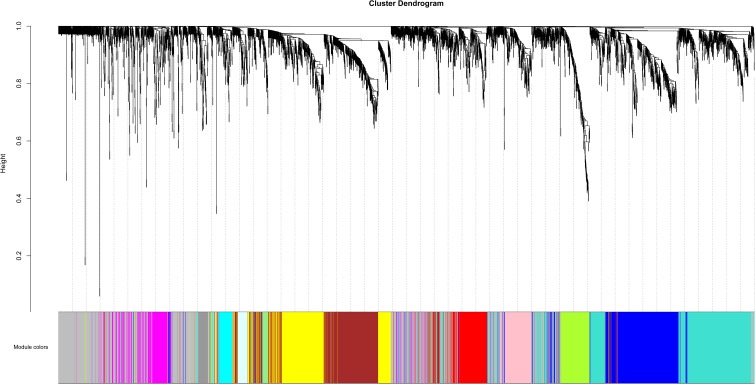
Clustering dendrogram of genes. The color bands provide a simple visual comparison of module assignments (branch cuttings) based on the dynamic tree cutting method.

Based on variance analysis, the top 25% of genes (5439 genes) was obtained from GSE51588 with 50 samples. As shown in Figure 1, the GSM1248767 sample was outlying. In the current study, the soft-thresholding parameter was determined as β = 12 where the curve first reached Rˆ^2^ = 0.85, to construct a weighted network based on a scale-free topology criterion (Figure 2). As is shown in Figure 3, 19 modules were detected through the dynamic tree cutting method.

### Identification of Clinically Significant Modules and Functional Annotation

**FIGURE 4:**
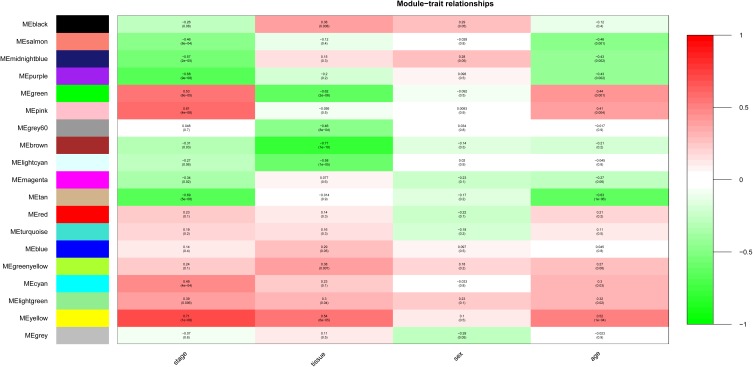
Module-trait relationships. Stage indicates OA and non-OA; tissue indicates samples from LT and MT.

**FIGURE 5:**
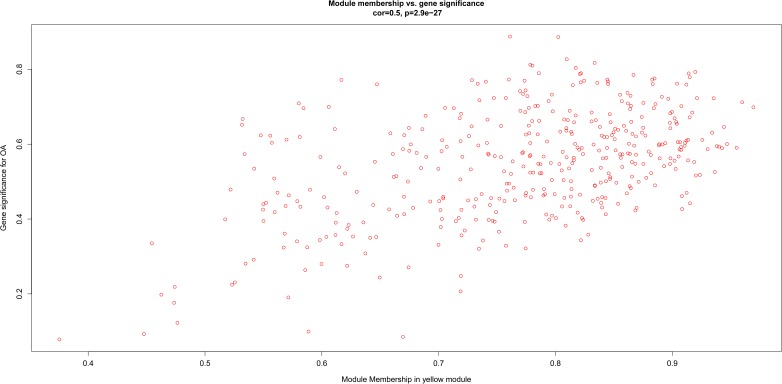
Scatter diagram for module membership vs. gene significance of stage (OA or non-OA) in yellow module.

**FIGURE 6:**
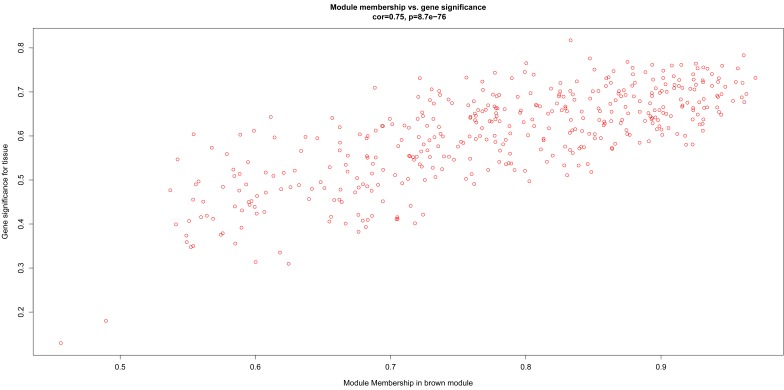
Scatter diagram for module membership vs. gene significance of tissue (LT or MT) in brown module.

After relating modules to traits, high correlations were observed in the trait of stage (OA or non-OA) and tissue (lateral tibial plateau and medial tibial plateau) (Figure 4). In terms of the trait of stage, the highest correlation was observed in the yellow-stage module (*r* = 0.71, *p* = 1e-08). As for tissue, the brown tissue module had the highest correlation (*r* = 0.77, *p* = 1e-10). Genes in the yellow (cor = 0.5, *p* = 2.9e-27, Figure 5) and brown modules (cor = 0.75, *p* = 8.7e-76, Figure 6) were characterized with high gene significance and module membership, based on an intramodular analysis. Supplementary Table 1 demonstrates that the yellow module was enriched in a variety of components including proteinaceous extracellular matrix, collagen trimers, endoplasmic reticulum lumen, extracellular regions, collagen catabolic processes, and components involved in osteoblast differentiation, etc., based on GO analysis (*p* < 0.001). Supplementary Table 2 shows that the yellow module included genes involved in protein digestion and absorption, axon guidance, ECM-receptor interaction, and the PI3K-Akt signaling pathway, etc. based on KEGG pathway analysis (*p* < 0.05). The brown module was enriched in components involved in extracellular space, extracellular region, extracellular matrix, cell adhesion, and brown fat cell differentiation, etc. (*p* < 0.001, Supplementary Table 3). In addition, the brown module contained components involved in tyrosine metabolism, drug metabolism (cytochrome P450), ECM-receptor interaction, and the regulation of lipolysis in adipocytes, etc. (*p* < 0.01, Supplementary Table 4). Finally, the yellow module, with 409 genes, and the brown module with 413 genes were deemed as clinically significant modules with associations with OA development.

### Identification and Validation of Hub Genes

Based on an absolute value of the geneModuleMembership > 0.9 and geneTraitSignificance > 0.2, 45 hub genes and 72 hub genes were selected from the yellow module and brown module, respectively (Tables 1, 2). These hub genes were mapped into the PPI network. As shown in Table 1 and Supplementary Figure 1, five common genes (COL24A1, COL5A2, COL3A1, MMP2, and COL6A1) were observed in the yellow module and PPI network associated with OA stage. Eight common genes (LIPE, LPL, LEP, SLC2A4, FABP4, ADH1B, ALDH4A1, and ADIPOQ) were observed in the brown module and PPI network associated with OA tissue (Table 2 and Supplementary Figure 2). The results of the validation of the hub genes from the yellow and brown modules are displayed in Supplementary Table 5.

**Table 1:** Hub genes from the yellow module.

Probe	Gene	GeneModuleMembership	Hub gene in the PPI network
A_23_P253958	LRRC17	0.969473235	No
A_23_P48198	GLT8D2	0.959877328	No
A_23_P74701	COL24A1	0.955163023	Yes
A_23_P56746	FAP	0.946811157	No
A_33_P3364741	MRC2	0.944254916	No
A_23_P33196	COL5A2	0.942647924	Yes
A_24_P211565	C1QTNF6	0.940056543	No
A_33_P3336700	SHROOM3	0.938124468	No
A_33_P3222917	CD276	0.936416148	No
A_33_P3380529	PRTFDC1	0.935426537	No
A_33_P3280845	THY1	0.934100495	No
A_24_P935491	COL3A1	0.930260322	Yes
A_23_P63432	RHBDL2	0.925783098	No
A_33_P3237135	MMP2	0.923664358	Yes
A_33_P3336686	CLIC3	0.921127157	No
A_33_P3260575	CERCAM	0.920273281	No
A_33_P3398156	CYS1	0.919702463	No
A_23_P99906	HOMER2	0.918751881	No
A_23_P151529	C14orf132	0.917087409	No
A_23_P130194	PYCR1	0.91518196	No
A_23_P52336	UNC5B	0.914926299	No
A_24_P167654	SLC8A3	0.91488311	No
A_33_P3309551	PTPRD	0.91401164	No
A_24_P74070	PARD6G	0.913560661	No
A_23_P302787	LOC375295	0.913478286	No
A_23_P111888	CTHRC1	0.912267336	No
A_24_P118196	GXYLT2	0.912119625	No
A_32_P29118	SEMA3D	0.912067684	No
A_32_P32254	COL6A1	0.911032192	Yes
A_33_P3360540	AGPAT2	0.910588547	No
A_24_P408736	GALNT5	0.908471069	No
A_23_P211504	KDELR3	0.90828204	No
A_23_P109171	BFSP1	0.907830969	No
A_23_P251499	PCOLCE	0.907617452	No
A_23_P101093	COPZ2	0.907504995	No
A_24_P227927	IL21R	0.906686906	No
A_23_P69586	FAT1	0.906224666	No
A_33_P3214159	CDH2	0.905437519	No
A_24_P827037	LRRC15	0.90472131	No
A_33_P3345041	FLJ32063	0.904134402	No
A_24_P215765	ATP10A	0.903870696	No
A_23_P159907	MAGED4B	0.903422964	No
A_24_P97825	CCDC69	0.902890892	No
A_23_P163567	SMPD3	0.901995577	No
A_23_P53193	SYTL2	0.901980687	No


**Table 2:** Hub genes from the brown module.

Probe	Gene	GeneModuleMembership	Hub gene in the PPI network
A_23_P151232	TMEM132C	0.969856283	No
A_33_P3242543	MAOA	0.963154509	No
A_33_P3293362	1-Mar	0.961606389	No
A_23_P64617	FZD4	0.961128123	No
A_33_P3294986	LIPE	0.960301918	Yes
A_23_P146233	LPL	0.959713729	Yes
A_23_P308058	TUSC5	0.956794965	No
A_33_P3371115	AQP7P3	0.955308305	No
A_24_P397817	LEP	0.95325835	Yes
A_23_P39251	PLIN5	0.947555058	No
A_23_P145786	MLXIPL	0.945273946	No
A_33_P3240018	PDE3B	0.944749134	No
A_23_P376704	CIDEA	0.944504899	No
A_23_P36658	MGST1	0.942865498	No
A_23_P158041	AQP7	0.942784686	No
A_23_P26154	PLIN1	0.942169131	No
A_23_P426305	AOC3	0.942153425	No
A_24_P484797	CIDECP	0.941562321	No
A_24_P154037	IRS2	0.941550679	No
A_24_P224727	CEBPA	0.93772696	No
A_23_P111402	RSPO3	0.937652396	No
A_23_P128084	ITGA7	0.934366709	No
A_33_P3214466	MESP1	0.934126996	No
A_23_P204736	GPD1	0.931926582	No
A_33_P3400763	PLIN4	0.931566336	No
A_33_P3405728	PKP2	0.931320128	No
A_23_P77493	TUBB3	0.931281047	No
A_32_P57810	RNF157	0.93118069	No
A_24_P291658	ADH1A	0.930900109	No
A_24_P206776	CRYAB	0.930810728	No
A_23_P381172	MRAP	0.9307345	No
A_23_P42975	PRKAR2B	0.928552349	No
A_23_P64721	HCAR3	0.928283637	No
A_23_P92025	CIDEC	0.927149576	No
A_23_P85015	MAOB	0.927020575	No
A_33_P3210488	COL6A3	0.925880431	No
A_23_P79978	SLC24A3	0.925824615	No
A_32_P40288	TMEM200A	0.92472156	No
A_33_P3275702	FMO2	0.924148077	No
A_23_P74609	G0S2	0.923837665	No
A_33_P3216933	SIK2	0.923807734	No
A_23_P125505	PPEF1	0.92380506	No
A_32_P151263	SLC2A4	0.923412235	Yes
A_23_P8820	FABP4	0.923354566	Yes
A_32_P33114	KLB	0.921659613	No
A_23_P386942	DIRAS1	0.919487688	No
A_33_P3353737	ADH1B	0.918564725	Yes
A_23_P81158	ADH1C	0.917832335	No
A_23_P21324	TWIST2	0.915863824	No
A_23_P145965	TPST1	0.91578472	No
A_33_P3401243	OLFML2B	0.915629239	No
A_23_P170337	ALDH4A1	0.915018976	Yes
A_23_P101131	GRP	0.914667348	No
A_23_P134237	RARRES2	0.913437641	No
A_23_P32165	LHX2	0.913193791	No
A_23_P408249	PCK1	0.912022648	No
A_23_P63736	LOC84856	0.909998126	No
A_23_P253029	BOK	0.909722845	No
A_23_P55477	ADORA2B	0.909432496	No
A_23_P109636	LRIG1	0.908001504	No
A_23_P15876	ALPK2	0.907280117	No
A_33_P3350374	C10orf58	0.905537789	No
A_23_P37892	GPT2	0.904170892	No
A_23_P88404	TGFB3	0.903958589	No
A_23_P20443	LZTS1	0.903319664	No
A_33_P3298216	MYO16	0.901721107	No
A_23_P164047	MMD	0.901691839	No
A_23_P72668	SDPR	0.901641073	No
A_23_P101407	C3	0.901610377	No
A_23_P369237	ADIPOQ	0.900895088	Yes
A_24_P213950	HEPACAM	0.900550393	No
A_23_P108075	SLC7A10	0.900280812	No


## Discussion

Because of the high rates of disability, low quality of life, major pain and the resulting huge economic burden caused by OA (Neogi and Zhang, 2013), a thorough study of OA, including risk factors, pathological processes, clinical manifestation, diagnosis, treatment, and prevention is particularly essential. In the current study, the WGCNA algorithm (Langfelder and Horvath, 2008) was adopted to explore OA biomarkers and pathological processes in samples of subchondral bone from OA and non-OA subjects.

After data set preprocessing, weighted gene network construction, and module identification, relating modules to traits and functional enrichment, we found that both the yellow and brown modules were associated with the occurrence of OA. Specifically, the yellow module with 45 hub genes played a key role in proteinaceous extracellular matrix, collagen trimers, and collagen catabolic processes, etc. and was highly associated with the formation of extracellular matrix and the development of the skeletal system. Extracellular matrix and collagen secreted by mesenchymal cells in subchondral bone (Matyas et al., 1997) are important components of cartilage and protect the articular surface from destruction in the early stage of OA (Sanchez et al., 2005). In addition, collagen is also a major component of bone. Therefore, collagen dysfunction could lead to bone and joint disease including OA.COL24A1, COL5A2, COL3A1, COL6A1 are members of the collagen gene family. COL24A1 was reported to play an important role in osteoblast differentiation and bone formation (Matsuo et al., 2008) through theTGF-β/Smads signaling pathway (Wang et al., 2012). To the best of our knowledge, COL5A2 has been associated with ischemic heart disease (Azuaje et al., 2013) and the development of cancers, such as bladder cancer (Li et al., 2017), glioblastoma (Vastrad et al., 2017), and gastric cancer (Cao et al., 2018), with no evidence for an association with OA. A similar role has been reported for COL3A1 (Yuan et al., 2017). COL6A1 was demonstrated as a hub gene in OA in a recent study (Guo et al., 2018). In the current study, the yellow module contained components involved in protein digestion and absorption, axon guidance, ECM-receptor interaction and the PI3K-Akt signaling pathway. The aforementioned four hub genes also identified in the PPI network could be regarded as real hub genes associated with OA through the ECM-receptor interaction and interaction with the PI3K-Akt signaling pathway.

MMP2, a common component of the yellow and PPI network encodes matrix metallopeptidase 2, which is released by inflammatory cells, contributing to the initiation and progression of OA (Alunno et al., 2017). Inflammation could directly affect synovial cells and chondrocytes through cytokines which would interfere with the repair of cartilage in OA patients.

To identify the difference between early-stage (lateral tibia) and end-stage (medial tibia) OA, we related the modules to tissue traits and the brown module with 72 hub genes was found to contain genes involved in extracellular processes and lipid metabolism. The GO analysis of the brown module demonstrated enrichment in components involved in the extracellular space, region and matrix, further demonstrating that the destruction of the extracellular matrix leads to the progression of OA. LIPE, LPL, LEP, SLC2A4, FABP4, ADH1B, ALDH4A1, and ADIPOQ are all involved in energy metabolism in subchondral bone cells. It was speculated that end-stage OA was associated with lower energy metabolism, compared with early-stage OA. Therefore, drugs to improve lipid metabolism could prevent OA progression.

The results of the validation demonstrated that COL5A2, COL3A1, and COL6A1 at the core of the yellow module and PPI network were associated with OA in samples collected from cartilage and subchondral bone. Other hub genes validated in the yellow module, including FAP, CD276, PRTFDC1, THY1, RHBDL2, CLIC3, CERCAM, CYS1, and HOMER2 had significant differences between OA and non-OA subjects and CD276 has been reported as a biomarker for OA in a previous study (Mobasheri and Henrotin, 2015). In the brown module, significant validation results were observed for FZD4, TUSC5, PDE3B, LIPE, LEP and SLC2A4 genes, of which, LIPE, LEP, and SLC2A4 were at the core of the module and PPI network. Although several hub genes did not demonstrate significant results in the GSE57218 validation, it is noted that these hub genes are still potentially associated with OA, because GSE57218 was obtained using cartilage samples rather than subchondral bone.

There are several highlights in the current study. Firstly, previous studies explored OA pathological processes and biomarkers using cartilage samples. Currently, few studies have focused on subchondral bone. An expression profile of subchondral bone could contribute to a comprehensive understanding of OA and provide more evidence to elucidate the initiation site of OA (bone or cartilage). Secondly, WGCNA has a particular advantage in processing gene expression datasets and the results of this study not only confirmed the findings of previous studies, but also provided new biomarkers for the further study of OA. However, the current study also possesses several limitations. Cartilage samples were used in the validation of hub genes in this study. These hub genes remain to be validated using subchondral bone samples in further studies.

## Conclusion

In the current study, we applied the WGCNA algorithm to process gene expression datasets and devised a yellow module with 45 hub genes and a brown module with 72 hub genes associated with OA. Compared with early-stage OA, the brown module revealed that the subchondral bone cells of end-stage OA had lower lipid metabolism. Drugs designed to target these hub genes could prevent the progression of OA.

## Author Contributions

H-YG, R-XW, CZ, and MY conceived and designed the study. R-XW, H-YG, L-LL, and YL performed the analysis procedures. JG, R-XW, L-LL, CZ, and YL analyzed the results. YL, L-LL, MY, and JG contributed to analysis tools. R-XW, H-YG, and CZ contributed to the writing of the manuscript. All authors reviewed the manuscript.

## Conflict of Interest Statement

The authors declare that the research was conducted in the absence of any commercial or financial relationships that could be construed as a potential conflict of interest.

## Footnotes

1 www.ncbi.nlm.nih.gov/geo/
