# Supplementary material for: Identification of the Biomarkers and Pathological Process of Osteoarthritis: Weighted Gene Co-expression Network Analysis
Source: Front Physiol. 2019 Mar 19;10:275. doi: 10.3389/fphys.2019.00275 (PMC6433881; doi:10.3389/fphys.2019.00275)

**Legends of Supplementary Figures and Tables**

**Supplementary Table 1 Gene ontology functional annotation analysis of the yellow module**

**Supplementary Table 2 Kyoto encyclopedia of genes and genomes pathway enrichment from the yellow module**

**Supplementary Table 3 Gene ontology functional annotation analysis from the brown module**

**Supplementary Table 4 Kyoto encyclopedia of genes and genomes pathway enrichment from the brown module**

**Supplementary Table 5** V**alidation of hub genes from yellow and brown module**

**Supplementary Figure 1 protein-protein interaction network of genes for the yellow module**

Note: Genes with more nodes were highlighted by the yellow color. The edge width was proportional to the score of protein-protein interaction based on the STRING database.

**Supplementary Figure 2 protein-protein interaction network of genes for the brown module**

Note: Genes with more nodes were highlighted by the yellow color. The edge width was proportional to the score of protein-protein interaction based on the STRING database.

**Supplementary Table 1** Gene ontology functional annotation analysis of the yellow module

| **Term** | **PValue** | **Genes** |
| --- | --- | --- |
| GO:0005578~proteinaceous extracellular matrix | 4.47E-23 | ALPL, ASPN, WNT5A, CTHRC1, PODNL1, MAMDC2, ADAMTS14, ADAMTSL2, LUM, SPOCK1, MMP2, CPZ, NOV, WNT1, WISP1, EMID1, COL6A2, PHOSPHO1, LOX, COL11A1, DPT, COL10A1, ANGPTL4, FLRT3, WNT10B, BMP1, PTPRZ1, COL22A1, COL15A1, SPARC, COL5A2, SLIT2, MMP11, SLIT3, ADAMTS6, OMD, BGN, MMP23B, COL1A2, COL24A1 |
| GO:0005581~collagen trimer | 2.75E-18 | CTHRC1, COL13A1, COL3A1, COL22A1, COL15A1, COL2A1, COLEC12, SERPINH1, COL5A2, PCOLCE, C1QTNF6, C1QTNF3, FCN2, EMID1, COL1A2, COL6A2, COL12A1, COL6A1, SCARA3, LOX, COL24A1, COL11A1, COL10A1 |
| GO:0005788~endoplasmic reticulum lumen | 6.16E-15 | WNT5A, ARSE, COL3A1, ARSI, COL2A1, SERPINH1, WNT1, KDELC1, P4HA2, P4HA3, COL6A2, COL12A1, COL6A1, GPX8, PDGFD, COL8A1, COL11A1, COL10A1, COL13A1, COL22A1, COL15A1, CERCAM, COL5A2, COL1A2, LIPC, FKBP10, COL24A1 |
| GO:0005576~extracellular region | 1.71E-13 | GDF6, ARSI, MMRN1, MMP2, NOV, AZGP1, WNT1, CGREF1, ISG15, APOD, HTRA1, SEMA3E, GPX3, COL12A1, LBP, LOX, PDGFD, COL11A1, PRSS35, COL10A1, GHR, HHIPL1, WNT10B, MATN4, COL22A1, SLIT2, CD163, SLIT3, MMP11, INHBB, BGN, UCN2, SERPINF1, PDGFRL, COL1A2, COL24A1, WNT5A, ENPP6, ACHE, FGFR3, ADAMTS14, LUM, CXCL3, COL3A1, PAMR1, COL2A1, EPHB3, ISM1, ITGBL1, FNDC5, CCL23, CRISPLD1, FCN2, FNDC1, COL6A2, COL6A1, COL8A1, ANGPTL5, SPP1, ANGPTL4, BMP1, CFB, COL13A1, COL15A1, NID2, SPARC, COL5A2, HPR, EPHA3, DKK3, OMD, UACA, PENK, LCN6, LIPC, BMP7, BMP8A |
| GO:0030574~collagen catabolic process | 7.19E-13 | ADAMTS14, COL13A1, COL3A1, MRC2, COL15A1, COL2A1, COL5A2, MMP2, MMP11, COL1A2, COL6A2, COL12A1, COL6A1, COL8A1, COL11A1, COL10A1 |
| GO:0005615~extracellular space | 5.42E-12 | S100A4, CTHRC1, GDF6, LRRC17, MMP2, AZGP1, WNT1, WISP1, APOD, HTRA1, FAP, SEMA3E, GPX3, SEMA3D, COL12A1, LOXL4, LBP, LOX, PDGFD, GHR, WNT10B, CPXM2, SLIT2, SLIT3, INHBB, STOM, MSMP, UCN2, CBLN2, SERPINF1, CPE, COL1A2, WFDC1, ALPL, WNT5A, ACHE, LUM, CXCL3, COL3A1, COL2A1, SPOCK1, SERPINH1, PCOLCE, CPZ, CCL23, C1QTNF6, C1QTNF3, COL6A2, VWC2, PTN, ANGPTL1, DPT, SPP1, ANGPTL4, FLRT3, BMP1, CFB, COL15A1, SPARC, DKK3, CDH13, OMD, C3ORF58, LIPC, BMP7, BMP8A |
| GO:0030199~collagen fibril organization | 9.88E-11 | ADAMTS14, LUM, COL3A1, COL1A2, COL12A1, COL2A1, LOX, COL11A1, SERPINH1, COL5A2, MMP11, DPT |
| GO:0031012~extracellular matrix | 7.62E-10 | ASPN, LUM, COL3A1, COL2A1, EDIL3, MMRN1, MMP2, PCOLCE, NOV, HTRA1, SBSN, COL6A2, COL6A1, COL12A1, COL8A1, DPT, COL15A1, CPXM2, NID2, COL5A2, MMP11, BGN, SERPINF1, MMP23B, COL1A2, BMP7 |
| GO:0030198~extracellular matrix organization | 3.68E-08 | MATN4, ADAMTSL2, COL13A1, LUM, COL3A1, COL2A1, SPARC, NID2, COL5A2, BGN, KAZALD1, COL1A2, COL6A2, COL6A1, LOX, COL8A1, COL11A1, SPP1, COL10A1 |
| GO:0001501~skeletal system development | 5.04E-08 | ALPL, BMP1, ARSE, FGFR3, SOX11, COL3A1, COL2A1, COL5A2, SHOX2, COL1A2, NKX3-2, COL12A1, ALX4, BMP7, PHEX, COL10A1 |
| GO:0007155~cell adhesion | 5.61E-08 | ACHE, OPCML, SPOCK1, CDH2, EDIL3, MMRN1, ITGBL1, SEMA5A, AZGP1, CGREF1, WISP1, FAT1, FAP, COL6A2, COL6A1, COL12A1, COL8A1, SPP1, DPT, COL15A1, NID2, CERCAM, PRPH2, THY1, EPHA3, CDH13, OMD, CDH15, HES5 |
| GO:0001503~ossification | 2.62E-06 | BMP1, KAZALD1, LRRC17, RIPPLY2, SPARC, BMP7, TMEM119, COL11A1, COL5A2, SPP1, BMP8A |
| GO:0005518~collagen binding | 1.12E-05 | ASPN, ACHE, LUM, MRC2, NID2, SPARC, LRRC15, SERPINH1, PCOLCE |
| GO:0005201~extracellular matrix structural constituent | 2.56E-05 | BGN, LUM, COL3A1, COL1A2, COL15A1, COL2A1, COL24A1, COL11A1, COL5A2 |
| GO:0071230~cellular response to amino acid stimulus | 2.68E-05 | COL3A1, COL1A2, COL6A1, CPEB1, PDGFD, SESN1, MMP2, COL5A2 |
| GO:0050919~negative chemotaxis | 3.78E-05 | SEMA5A, FLRT3, SEMA5B, SEMA3E, SEMA3D, SLIT2, SLIT3 |
| GO:0001958~endochondral ossification | 1.12E-04 | ALPL, FGFR3, COL13A1, PHOSPHO1, COL2A1, COL10A1 |
| GO:0070062~extracellular exosome | 1.14E-04 | S100A4, SLC36A2, ARSE, RARRES1, OPCML, CHMP4C, SLC20A2, LXN, GREB1, EDIL3, LRRC15, ALDH1L2, EPCAM, AZGP1, WNT1, APOD, HTRA1, SBSN, GPX3, COL12A1, LOXL4, LBP, PDGFD, GNG4, CYS1, LAD1, ACO1, CDHR2, LIFR, CYB5A, COLEC12, SLIT2, THY1, STOM, FOLH1, BGN, SERPINF1, CPE, CLIC3, COL1A2, ALPL, WNT5A, TPPP3, ENPP6, SHROOM2, FOLH1B, LUM, CDH2, SERPINH1, PCOLCE, CPZ, SEMA5A, PFN2, CRISPLD1, C1QTNF3, FAT1, FCN2, COL6A2, FASN, COL6A1, ANGPTL1, COL8A1, DPT, SPP1, SHMT1, PTPRD, CFB, MYO1G, CD276, COL15A1, NID2, HPR, SOD2, LIN7A, ABCB4, XPNPEP2, CDH13, OMD, UACA, CDH15, HEBP2, PPIC |
| GO:0051216~cartilage development | 1.20E-04 | WNT5A, SATB2, HES5, LUM, COL2A1, BMP7, COL10A1, BMP8A |
| GO:0035987~endodermal cell differentiation | 1.35E-04 | COL12A1, COL6A1, HMGA2, COL8A1, COL11A1, MMP2 |
| GO:0005886~plasma membrane | 1.82E-04 | SLC8A3, SLC36A2, SLC13A5, SGMS2, SLC44A3, PLXNA2, ADCY5, SLC9A2, TLR2, LPAR4, LPAR3, MMP2, GLDC, AZGP1, WNT1, UNC5B, HTRA1, FAP, SLCO1C1, GNG4, SLC4A3, C5AR1, LIFR, COLEC12, CERCAM, THY1, CPE, IGSF3, PTGFRN, WNT5A, ENPP6, ACHE, FGFR3, FOLH1B, MRAP2, EPHB3, P2RY8, KCNS3, SEMA5A, KCNS1, ENTPD3, MRC1, CFB, BFSP1, MYO1G, HOMER2, ABCG4, ABCB4, LIN7A, EPHA3, KCNK4, PENK, PKP3, SYT13, SYTL2, RHBDL2, CACNA1E, PARD6G, PHEX, TMEM119, IGDCC4, OPCML, SLC20A2, ATP10A, FCRL2, RHOU, EPCAM, SLC1A3, GUCY1A2, STRA6, ANO5, GHR, SHC4, RFTN2, SLC7A11, SLIT2, CD163, FOLH1, STXBP6, SGCD, ALPL, SHROOM2, FPR1, OXTR, CDH2, OR10G2, OR10G3, LINGO1, APLNR, FNDC5, ACSL1, FAT1, FASN, PRSS12, B4GALNT1, FLRT3, PTPRD, COL13A1, FZD1, SPARC, DGKI, C9ORF3, XPNPEP2, CDH13, CDH15, SCN4B, BAMBI, HTR2B, SMPD3, HTR2A |
| GO:0007507~heart development | 1.97E-04 | FLRT3, COL3A1, PPARG, OXTR, SPARC, SOD2, SHOX2, APLNR, SALL1, PTN, STRA6, LOX, HTR2B |
| GO:0005109~frizzled binding | 4.31E-04 | WNT5A, CTHRC1, WNT1, WNT10B, FZD1, BAMBI |
| GO:0008201~heparin binding | 6.37E-04 | NOV, CCL23, WISP1, LXN, COL13A1, PTN, BMP7, LIPC, SLIT2, PCOLCE, SLIT3 |
| GO:0060411~cardiac septum morphogenesis | 7.31E-04 | DHRS3, HEY1, HEY2, BMP7 |
| GO:0002062~chondrocyte differentiation | 8.01E-04 | NOV, WNT10B, FGFR3, HES5, COL2A1, HMGA2 |
| GO:0001649~osteoblast differentiation | 8.08E-04 | SHOX2, ALPL, PENK, MRC2, FASN, COL6A1, LRRC17, TMEM119, SPP1 |
| GO:0048407~platelet-derived growth factor binding | 8.49E-04 | COL3A1, COL1A2, COL6A1, COL2A1 |
| GO:0046426~negative regulation of JAK-STAT cascade | 9.02E-04 | ASPN, FLRT3, BGN, PODNL1, HMGA2, LRRC15 |
| GO:0004222~metalloendopeptidase activity | 9.85E-04 | ADAMTS6, BMP1, ADAMTS14, ADAMTSL2, MMP23B, FAP, PHEX, MMP2, MMP11 |

**Note:** GO, gene ontology; P< 0.001

**Supplementary Table 2** Kyoto encyclopedia of genes and genomes pathway enrichment from the yellow module

| **Term** | **PValue** | **Genes** |
| --- | --- | --- |
| hsa04974:Protein digestion and absorption | 4.54E-09 | SLC8A3, COL13A1, COL3A1, COL22A1, COL15A1, COL2A1, COL5A2, XPNPEP2, COL1A2, COL6A2, COL12A1, COL6A1, COL24A1, COL11A1, COL10A1 |
| hsa04360:Axon guidance | 4.10E-04 | SEMA5A, SEMA5B, UNC5B, PLXNA2, ABLIM3, SEMA3E, SEMA3D, EPHB3, SLIT2, EPHA3, SLIT3 |
| hsa04512:ECM-receptor interaction | 5.61E-04 | COL3A1, COL1A2, COL6A2, COL6A1, COL2A1, COL24A1, COL11A1, COL5A2, SPP1 |
| hsa04151:PI3K-Akt signaling pathway | 0.00318819 | FGFR3, COL3A1, TLR2, LPAR4, LPAR3, COL2A1, COL5A2, COL6A2, COL1A2, GYS2, COL6A1, PDGFD, COL24A1, GNG4, COL11A1, GHR, SPP1 |
| hsa04510:Focal adhesion | 0.013910511 | COL3A1, COL1A2, COL6A2, COL6A1, COL2A1, PDGFD, COL24A1, COL11A1, COL5A2, SHC4, SPP1 |
| hsa03320:PPAR signaling pathway | 0.014799148 | CPT1C, ACSL1, SCD, PPARG, FADS2, ANGPTL4 |
| hsa01212:Fatty acid metabolism | 0.019815053 | CPT1C, ACSL1, SCD, FASN, FADS2 |
| hsa04611:Platelet activation | 0.022671463 | ADCY5, COL3A1, GUCY1A2, COL1A2, COL2A1, COL24A1, COL11A1, COL5A2 |
| hsa00330:Arginine and proline metabolism | 0.022689107 | PYCR1, CKM, P4HA2, P4HA3, SMOX |
| hsa05146:Amoebiasis | 0.027362449 | COL3A1, COL1A2, TLR2, COL2A1, COL24A1, COL11A1, COL5A2 |
| hsa00061:Fatty acid biosynthesis | 0.031214012 | ACSL1, OLAH, FASN |
| hsa04390:Hippo signaling pathway | 0.045663591 | WNT5A, WNT1, WNT10B, GDF6, FZD1, PARD6G, BMP7, BMP8A |

**Note:** P<0.05.

**Supplementary Table 3** Gene ontology functional annotation analysis from the brown module

| **Term** | **PValue** | **Genes** |
| --- | --- | --- |
| GO:0005615~extracellular space | 9.15E-08 | AEBP1, IGFBP6, TGFB3, POSTN, CXADR, IL11, ACTG2, SERPINE2, FRMD7, SEMA3G, CPA3, ANGPT1, CAT, CPA1, CFD, ANGPT2, EBI3, SPON1, RS1, CES1P1, SPARCL1, GNLY, TCN2, GRP, TNFAIP6, LILRB2, CD36, CPXM1, CSTA, EPYC, IBSP, RBP4, WNT16, C3, CD109, TIMP4, ALDH3A1, COL7A1, COL6A3, TFF3, KRT2, THBS4, COL18A1, LPL, KL, ECM1, ADIPOQ, LEP, LAMA1, CCL14, TNFSF11, S100B, CXCL13, SULF1, GPT, CP, TPSAB1, PLAU |
| GO:0005576~extracellular region | 2.92E-07 | RARRES2, IGFBP6, TGFB3, FGF13, CXADR, DNASE1L3, IL11, RSPO4, SERPINE2, RSPO3, ITIH5, CPA3, ANGPT1, CFD, FAM150B, PRL, ANGPT2, EBI3, MATN3, C2CD2, OLFML2B, TCN2, TPSD1, C1QA, GRP, INHBA, CHRDL1, MFAP2, ADAM12, TREM2, IBSP, C7, RBP4, WNT16, C3, C6, NMB, FAM19A5, COL9A1, COL7A1, COL6A3, TFF3, SCG5, THBS2, THBS4, COL18A1, LPL, KL, DMP1, LY6K, ECM1, ADIPOQ, LEP, VWF, LAMA1, NOTCH1, CCL14, TNFSF11, S100B, CXCL13, CP, TPSAB1, APOL6, PLAU |
| GO:0031012~extracellular matrix | 1.08E-05 | IBSP, COL18A1, LPL, RARRES2, AEBP1, TGFB3, POSTN, MMP14, ECM1, LAMA1, VWF, HMCN1, SERPINE2, COL7A1, COL6A3, CSTA, TPSAB1, THBS2, THBS4, SPON1 |
| GO:0007155~cell adhesion | 4.58E-05 | IBSP, COL18A1, F11R, MYBPC2, AMTN, ITGA11, POSTN, TPBG, LAMA1, TNFAIP6, VWF, LYVE1, SRPX, CD36, COL7A1, HEPACAM, COL6A3, ITGA7, GPNMB, ADAM12, THBS2, THBS4, RS1, AOC3, SPON1 |
| GO:0050873~brown fat cell differentiation | 4.87E-05 | CEBPA, RARRES2, ADRB1, SLC2A4, MRAP, FABP4, ADIPOQ |
| GO:0005811~lipid particle | 5.50E-05 | PLIN5, DGAT2, PLIN1, PLIN4, CIDEA, FABP4, G0S2, LIPE, CIDEC |
| GO:0055114~oxidation-reduction process | 6.37E-05 | TM7SF2, ALDH1L1, ADHFE1, BBOX1, ALDH3A1, AKR1C3, TDO2, AKR1C4, FMO3, ALDH4A1, NQO1, MAOA, MAOB, FADS3, ACADL, CYP4B1, RDH5, VAT1L, AKR1B15, NXN, DIO2, AKR1B10, HSD11B1, ASPHD1, CP, HPGD, MGST1, PRODH, AOC3 |
| GO:0030198~extracellular matrix organization | 7.57E-05 | IBSP, COL18A1, F11R, MATN3, OLFML2B, DMP1, ITGA11, POSTN, COL9A1, VWF, LAMA1, COL7A1, COL6A3, ITGA7, MFAP2 |
| GO:0005578~proteinaceous extracellular matrix | 1.25E-04 | COL18A1, MATN3, WNT16, AMTN, SPARCL1, OLFML2B, DMP1, TIMP4, POSTN, ECM1, COL9A1, VWF, LAMA1, COL6A3, TFF3, EPYC, SPON1 |
| GO:0005887~integral component of plasma membrane | 1.65E-04 | TM7SF2, LRRC8E, MCHR1, PLXNA4, GJA1, AQP7, CXADR, VIPR1, EDNRA, SLC1A5, SLC2A4, SLC24A3, SLC22A3, HCAR3, MLANA, EFNB3, SLC7A10, PCDH7, MMP14, LILRB2, ADRB1, CD36, CLDN1, ADORA2B, SYNDIG1, MMD, KCNA4, GPR1, SLCO2A1, ADRA2A, SCN9A, SCNN1B, GPNMB, GABRD, GABRA1, TRHDE, KLB, KL, GABRA6, TNFRSF13B, NPR3, KCNK1, FZD4, KCNK2, TPBG, FZD10, LYVE1, TNFSF11, SLC13A3, AQP7P3 |
| GO:0016491~oxidoreductase activity | 2.65E-04 | ALDH1L1, ADHFE1, MAOA, MAOB, FADS3, ADH1C, ADH1B, ALDH3A1, AKR1C3, VAT1L, HSD11B1, ALDH2, ALDH4A1, HPGD |
| GO:0042593~glucose homeostasis | 2.88E-04 | LEP, CEBPA, RBP4, SLC2A4, WFS1, ADRA2A, MLXIPL, NMB, ADIPOQ, PCK1 |
| GO:0016324~apical plasma membrane | 3.19E-04 | PARD6B, LZTS1, MAL2, KL, GJA1, CNTFR, KCNK1, KCNK2, NOTCH1, CD36, SLC29A4, TMEM114, CLDN1, CA4, SCNN1B, RAB27B, GPIHBP1 |
| GO:0030175~filopodium | 5.85E-04 | ACTG2, PPP1R9A, MYO3A, GPM6A, ERMN, FGF13, CXADR, FGD4 |

**Note:** GO, Gene ontology; P<0.001.

**Supplementary Table 4** Kyoto encyclopedia of genes and genomes pathway enrichment from the brown module

| **Term** | **PValue** | **Genes** |
| --- | --- | --- |
| hsa00350:Tyrosine metabolism | 1.40E-04 | MAOA, MAOB, ADH1C, ADH1B, ADH1A, ALDH3A1, AOC3 |
| hsa00982:Drug metabolism - cytochrome P450 | 1.72E-04 | FMO2, MAOA, FMO3, MAOB, ADH1C, ADH1B, ADH1A, MGST1, ALDH3A1 |
| hsa04512:ECM-receptor interaction | 1.84E-04 | IBSP, LAMA1, VWF, CD36, COL6A3, ITGA7, ITGA11, SV2B, THBS2, THBS4 |
| hsa03320:PPAR signaling pathway | 9.12E-04 | LPL, CD36, PLIN1, FABP4, AQP7, ACADL, ADIPOQ, PCK1 |
| hsa04923:Regulation of lipolysis in adipocytes | 0.001865 | IRS2, ADRB1, PLIN1, PDE3B, FABP4, AQP7, LIPE |
| hsa04974:Protein digestion and absorption | 0.004421 | COL18A1, SLC1A5, COL9A1, KCNN4, COL7A1, COL6A3, CPA3, CPA1 |
| hsa00010:Glycolysis / Gluconeogenesis | 0.00464 | ADH1C, ALDH2, HK2, ADH1B, ADH1A, PCK1, ALDH3A1 |
| hsa04610:Complement and coagulation cascades | 0.005364 | C1QA, C7, VWF, C3, C6, CFD, PLAU |
| hsa04920:Adipocytokine signaling pathway | 0.005756 | LEP, IRS2, CD36, SLC2A4, ACACB, ADIPOQ, PCK1 |
| hsa00360:Phenylalanine metabolism | 0.006754 | MAOA, MAOB, ALDH3A1, AOC3 |
| hsa00980:Metabolism of xenobiotics by cytochrome P450 | 0.007534 | AKR1C4, HSD11B1, ADH1C, ADH1B, ADH1A, MGST1, ALDH3A1 |
| hsa04152:AMPK signaling pathway | 0.007706 | PPP2R1B, LEP, IRS2, CD36, SLC2A4, PFKFB1, ADIPOQ, LIPE, PCK1 |

**Note:** P<0.01.

**Supplementary Table 5** Validation of hub genes from yellow and brown module

| **Genes from yellow module** | **P** |  | **Genes from brown module** | **P** |
| --- | --- | --- | --- | --- |
| LRRC17 | 0.185 |  | TMEM132C | 0.109 |
| GLT8D2 | 0.534 |  | MAOA | 0.596 |
| COL24A1 | 0.434 |  | 1-Mar | NA |
| FAP | 4.18E-07 |  | FZD4 | 0.0447 |
| MRC2 | 0.0742 |  | LIPE | 0.00727 |
| COL5A2 | 0.009 |  | LPL | 0.422 |
| C1QTNF6 | 0.0585 |  | TUSC5 | 0.0201 |
| SHROOM3 | NA |  | AQP7P3 | NA |
| CD276 | 0.0208 |  | LEP | 4.81E-06 |
| PRTFDC1 | 0.0208 |  | PLIN5 | NA |
| THY1 | 4.38E-07 |  | MLXIPL | 0.199 |
| COL3A1 | 0.00011 |  | PDE3B | 1.81E-07 |
| RHBDL2 | 6.28E-06 |  | CIDEA | 0.36 |
| MMP2 | 0.918 |  | MGST1 | 0.136 |
| CLIC3 | 2.12E-05 |  | SLC2A4 | 0.0234 |
| CERCAM | 0.0229 |  | FABP4 | 0.13 |
| CYS1 | 0.00592 |  | ADH1B | 0.567 |
| HOMER2 | 0.00913 |  | ALDH4A1 | 0.144 |
| COL6A1 | 0.009 |  | ADIPOQ | 0.223 |

**Note:** NA, Not available; P<0.05.

**Supplementary Figure 1 protein-protein interaction network of genes for the yellow module**


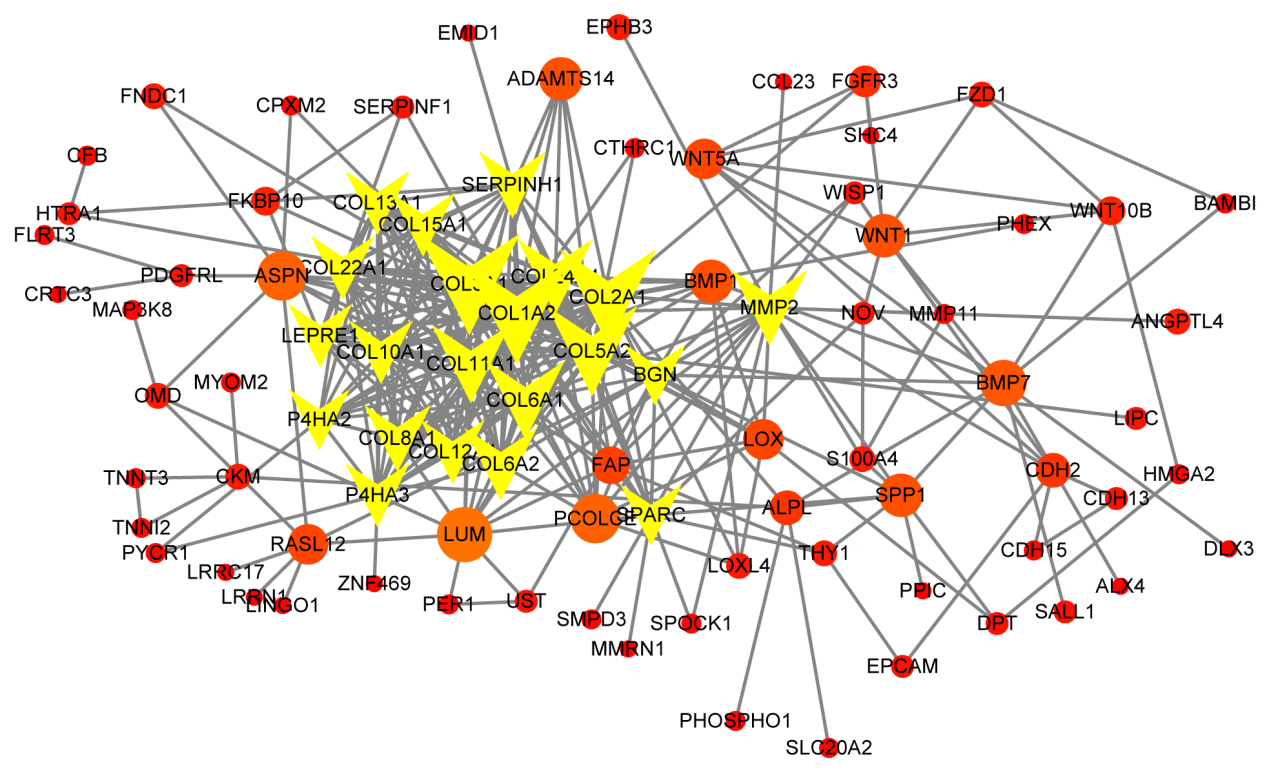


**Supplementary Figure 2 protein-protein interaction network of genes for the brown module**


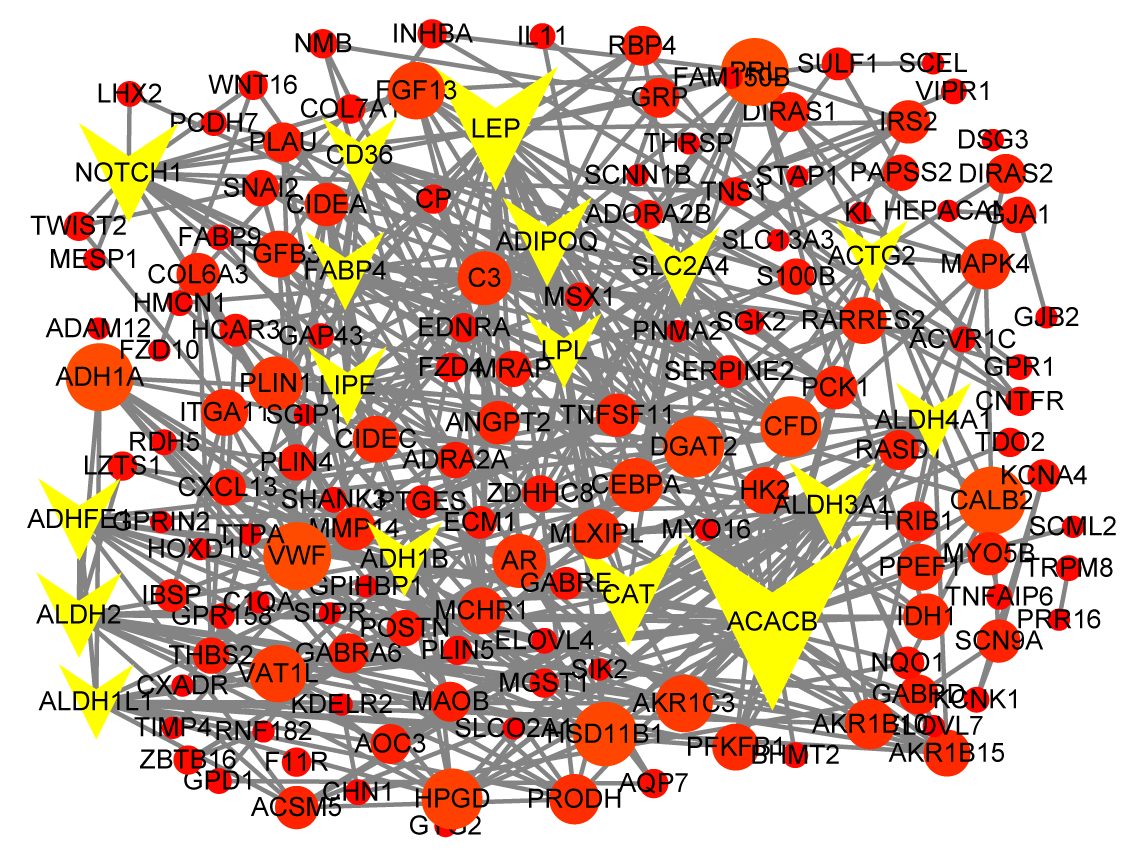

Supplement: Supplementary file 1 [file Table_1.docx]
